# The combination of lonafarnib and sorafenib induces cyclin D1 degradation via ATG3-mediated autophagic flux in hepatocellular carcinoma cells

**DOI:** 10.18632/aging.102165

**Published:** 2019-08-13

**Authors:** Jialiang Wang, Huan Wei, Yanlin Huang, Dongmei Chen, Guofen Zeng, Yifan Lian, Yuehua Huang

**Affiliations:** 1 Guangdong Provincial Key Laboratory of Liver Disease Research, The Third Affiliated Hospital of Sun Yat-sen University, Guangzhou, China; 2 Department of Infectious Diseases, The Third Affiliated Hospital of Sun Yat-sen University, Guangzhou, China

**Keywords:** lonafarnib, sorafenib, autophagy, DNA synthesis, hepatocellular carcinoma

## Abstract

Combination treatment is a promising strategy to improve prognosis of hepatocellular carcinoma (HCC). Sorafenib is a traditional first-line agent approved for the treatment of advanced HCC, though with limited efficacy. Previously, we reported that lonafarnib, an orally bioavailable non-peptide inhibitor targeting farnesyltransferase, synergizes with sorafenib against the growth of HCC cells. In the present study, we aim to clarify the underlying mechanism of this combination strategy. Initially, using in vitro HCC cell model, we confirmed that synergistic treatment of lonafarnib and sorafenib suppressed cell viability and colony formation, and induced cell death. We then found conversion of LC3-I to LC3-II via combination the treatment and observed formation of autophagosomes by electron microscopy. Knockdown of ATG3 inhibited the autophagic flux induced by the combination treatment. Furthermore, we demonstrated that drug-eliciting autophagy selectively promoted the degradation of cyclin D1 in a lysosome-dependent manner and subsequently inhibited DNA synthesis through downregulating the phosphorylation of Rb protein. In conclusion, our results provide a deeper insight into the mechanism for the combination treatment of lonafarnib and sorafenib in HCC therapy.

## INTRODUCTION

Liver cancer is one of the most prevalent malignancies and the second leading cause of cancer death due to its high recurrence rate and poor prognosis [1], estimated to have accounted for approximately 700,000 deaths in 2012 [2]. As the most common type of primary liver cancer, the incidence rate of hepatocellular carcinoma (HCC) accounts for 85%–90% of all cases [3]. In China, 18.43 per 10,000 patients are diagnosed with HCC annually [4]. Currently, curative treatments including liver transplantation and hepatic resection are only suitable for 20% of HCC patients at a very early stage and there is no adequate and effective therapy for patients with advanced, metastatic or drug-resistant HCC [5, 6]. Therefore, it is essential to clarify the mechanisms of tumorigenesis, metastasis, and drug resistance in HCC.

Although oncogenes and recurrent driver genes have been identified in HCC [7–10], most of them cannot yet be considered as druggable targets for effective and safe therapy. Sorafenib is one of the two first-line drugs approved by the FDA for the management and treatment of advanced HCC as an inhibitor of RAF-MEK-ERK/ MAPK pathway that suppresses tumor cell proliferation and induces apoptosis. This agent is beneficial to approximately 30% of HCC patients and extends the survival time by 3–5 months clinically. However, a considerable number of HCC patients are refractory to sorafenib due to acquired drug resistance. Moreover, only limited effect of sorafenib is observed when combined with transarterial chemoembolization (TACE), radiotherapy, and other traditional chemodrugs [11].

Lonafarnib is an orally active, potent and selective inhibitor targeting human farnesyltransferases (FTases) which are responsible for post-translational lipid modification of extensive cellular proteins that are involved in the pathogenic pathways of various diseases. *In vitro* and *in vivo* studies have revealed that lonafarnib monotherapy has remarkable effects on human tumor cell lines lacking Ras activity, including lung, pancreas, colon, prostate, urinary bladder and hematological malignancies [12, 13]. However, as a single agent, its therapeutic function is limited and could be improved by combination usage with cytotoxic drugs, radiation or other novel targeted chemicals. Although the roles of lonafarnib in clinical trials are controversial, it remains a promising antitumor agent [14].

Autophagy is a “self-degradative” programmed degradation mechanism by facilitating the engulfment and digestion of damaged, long-lived or misfolded proteins and organelles in response to environmental challenges. This process includes serial stage and sequential formation of autophagosomes, lysosome fusion and autolysosome degradation. Autophagosome formation requires concerted actions of a distinguished set of autophagy mediator proteins named ATG (autophagy-related) proteins. During these stages, two ubiquitin-like conjugation systems are included: the ATG5-ATG12-ATG16 conjugation system and the LC3 conjugation system. The LC3 proteins are first cleaved by ATG4 cysteine proteases and a glycine residue is exposed, becoming the free cytosolic LC3-I. Subsequently, LC3-I is converted to its lapidated form called LC3-II and localizes at the mature autophagosomes with the help of E1-like enzyme ATG7 and E2-like enzyme ATG3. After the establishment of autophagosome vesicles, LC3-II is incorporated into the autophagosomal membrane at the late stage [15]. Notably, ATG3 and ATG7 are indispensable for ATG8-phosphatidylethanolamine conjugation and are therefore essential for the autophagosome formation. Thus, clarifying the regulatory mechanism of ATG7 and ATG3 expressions may provide a better understanding of targeted autophagy therapy [16].

Cyclin D1 is overexpressed in HCC via gene amplification and remains as a risk factor for HCC occurrence [17, 18]. This protein is synthesized at G1 phase and degraded in the cytoplasm when cell cycle enters S phase [19, 20]. As a regulatory subunit of cyclin-dependent kinases (CDKs), cyclin D1 binds and activates CDK4/6 at G1 phase and then phosphorylates Retinoblastoma protein (Rb) to promote the G1/S phase transition [21]. Rb can inhibit gene transcription through binding to the transcription factor E2F1-3, and phosphorylated Rb releases E2Fs and transcriptionally initiates c-myc gene to accelerate cell cycle progression and promote DNA synthesis [22]. Thus, targeting cyclin D1 could be a way to treat HCC via regulating cell cycle progression.

In this study, we demonstrate that the combination of lonafarnib and sorafenib suppresses HCC cell growth and induces autophagic flux that selectively degrades cyclin D1 expression and subsequently inhibits DNA synthesis. Our study contributes to clarifying the underlying mechanism of the combination use of lonafarnib and sorafenib in HCC treatment.

## RESULTS

### Combination treatment of lonafarnib and sorafenib inhibits HepG2 cell growth

**Figure 1:**
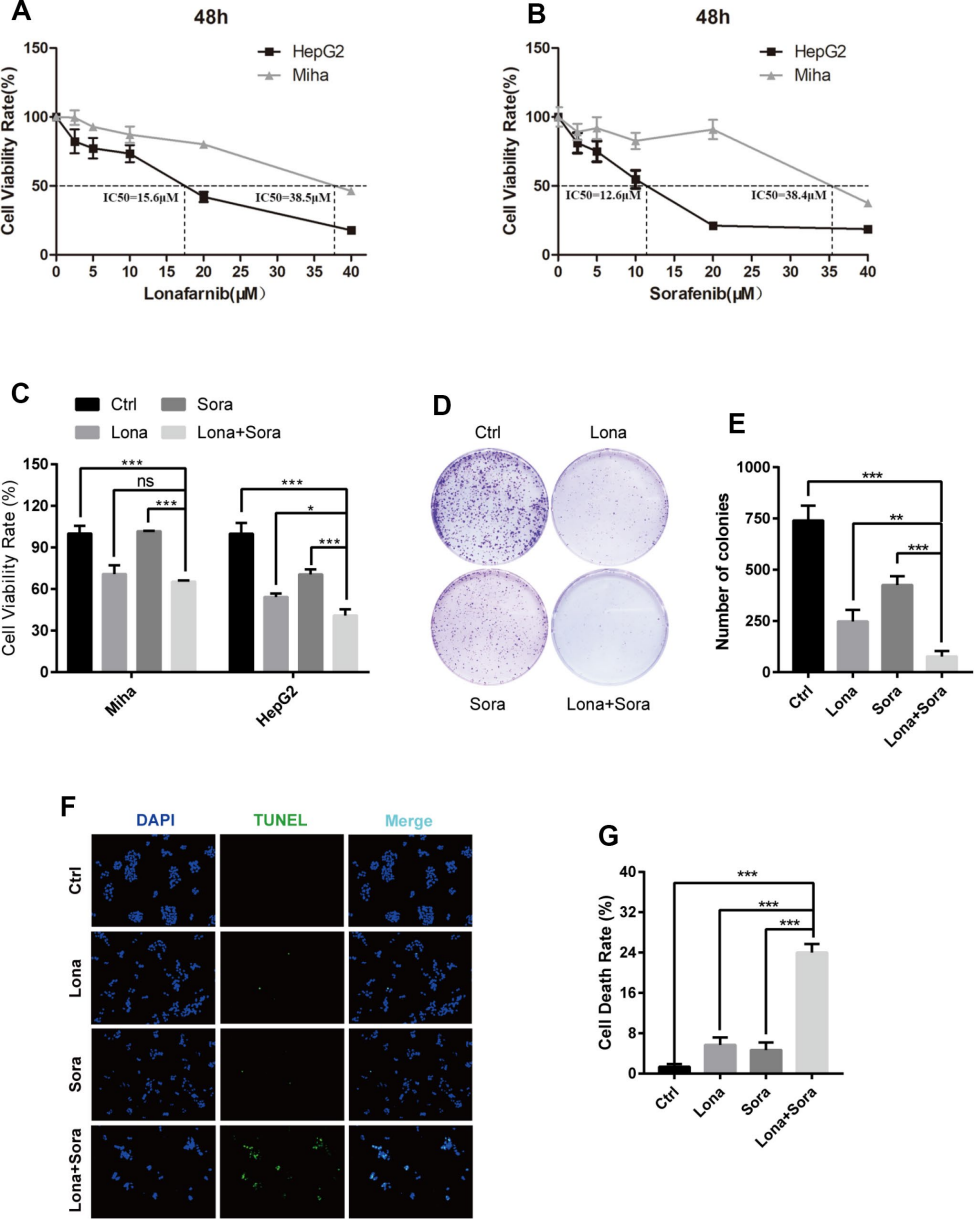
**Combination treatment of lonafarnib and sorafenib inhibits HepG2 cell growth.** (**A** and **B**) HepG2 and MIHA cells were subjected to CCK-8 assay with escalatory concentrations of lonafarnib or sorafenib. The IC50 value at 48 h was determined in these cell lines: HepG2 (lonafarnib: 15.6 μM, sorafenib: 12.6 μM), MIHA (lonafarnib: 38.5 μM, sorafenib: 38.4 μM). (**C**) Dose escalation effect of lonafarnib and sorafenib on the viability of HepG2 and MIHA cells measured at 48 h by CCK-8 assay. ns, P > 0.05; *P < 0.05; ***P < 0.001. (**D**) Colony formation assay in HepG2 cells. Cells were treated with lonafarnib (10 μM) and/or sorafenib (5 μM) for 14 days. At the end of this period, cells were stained with 0.5% crystal violet. (**E**) The number of colonies is calculated and presented as the means ± SD of triplicates. **P < 0.01; ***P < 0.001. (**F**) Representative image of TUNEL assay. HepG2 cells were maintained in 10 μM lonafarnib and/or 5 μM sorafenib for 48 h. The green puncta indicate the broken DNA fragment in cells. (**G**) The number of TUNEL-labeled DNA fragments were presented as the means ± SD of triplicates. ***P < 0.001.

We firstly performed CCK-8 assay to measure the monotherapy effect of lonafarnib or sorafenib alone on one immortalized liver cell line, MIHA, and one HCC cell line, HepG2, with different increasing concentrations. After 48 h of treatment, both of these drugs markedly suppressed the proliferation of HepG2 cells in a dose-dependent manner. The IC50 values were 15.6 μM for lonafarnib and 12.6 μM for sorafenib in HepG2 cells, respectively (Figure 1A and 1B). However, only limited growth inhibition was observed and the IC50 values were 38.5 μM for lonafarnib and 38.4 μM for sorafenib in MIHA cells, respectively (Figure 1A and 1B). According to the dose-response curves above, we used low and acceptable concentrations of lonafarnib (10 μM) and sorafenib (10 μM) for further single or combinatory treatment. We found that after 48 h of treatment, the combination group displayed a robust decrease in both HepG2 and MIHA cell viability compared to the single agent groups, but it is worth mentioning that HepG2 cells were more sensitive to the drug combination than MIHA cells (Figure 1C). In addition, combination treatment of lonafarnib and sorafenib significantly reduced colony formation in HepG2 cells as compared to the single agent groups (Figure 1D and 1E). Moreover, TUNEL assay showed that DNA damage-induced cell death was obviously increased in drug combination group (Figure 1F and 1G). These results demonstrate that the synergistic treatment of lonafarnib and sorafenib suppresses human HCC cell growth.

### Co-treatment of lonafarnib and sorafenib suppresses HCC cell growth *in vivo*

**Figure 2:**
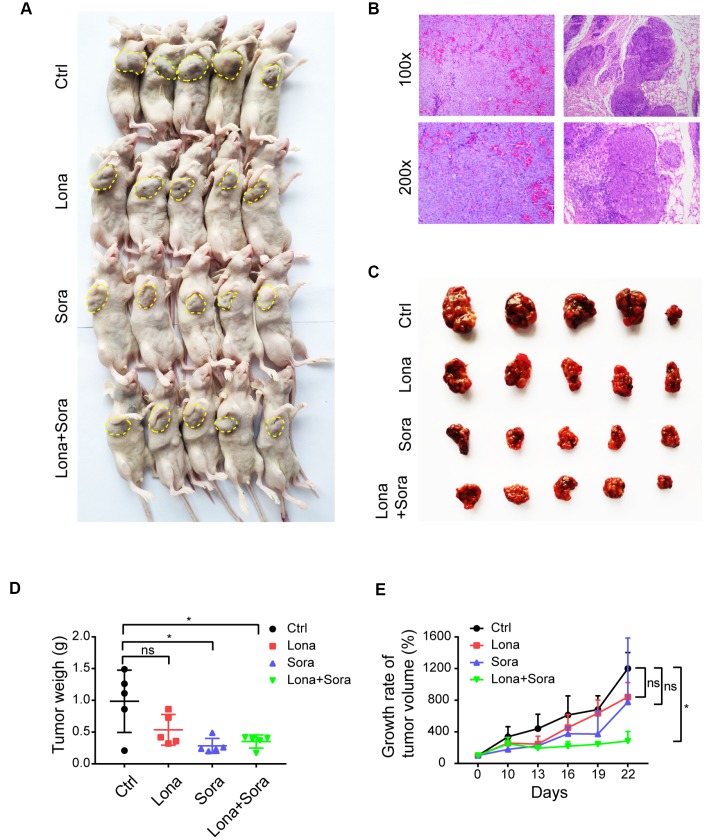
**Co-treatment of lonafarnib and sorafenib suppresses HCC cell growth *in vivo*.** (**A**) All mice used in the xenograft experiment were shown. The yellow dashed line depicts the border of palpable xenograft tumors. (**B**) Hematoxylin and eosin (**H** and **E**) staining of one xenograft tumor. (**C**) Isolated tumors from nude mice at the experimental endpoint. (**D**–**E**) Weights (**D**) and growth curves (**E**) of xenograft tumors from nude mice at the experimental endpoint. ns, P > 0.05; *P < 0.05.

Our *in vitro* study has demonstrated that lonafarnib and sorafenib combination therapy strongly suppressed HCC cell growth. We subsequently used the nude mice subcutaneous tumor model to evaluate the synergistic effect of these agents *in vivo*. Initially, all mice were injected with 1×10^7^ HepG2 cells and when palpable tumors started to form, mice were randomly assigned into four groups as indicated. As shown in Figure 2A and 2B, tumors were formed and grew in all mice after injection. Significant lower tumor weights were observed in combination group compared to control group (Figure 2C and 2D). However, the tumor weights did not show significant difference in combination group compared to monotherapy groups, neither in lonafarnib group compared to control group (Figure 2C and 2D). This may be due to the inconsistency of average tumor size at baseline. After data normalization, both single treatment of lonafarnib or sorafenib lead to slower tumor growth in vivo, though not reaching statistical significance (Figure 2E). Noticeably, greater and significant tumor suppression was observed in combination group compared to control group (Figure 2E). These results confirmed that co-treatment of lonafarnib and sorafenib inhibited HCC tumor growth effectively *in vivo*.

### Lonafarnib combined with sorafenib induces autophagic flux

**Figure 3:**
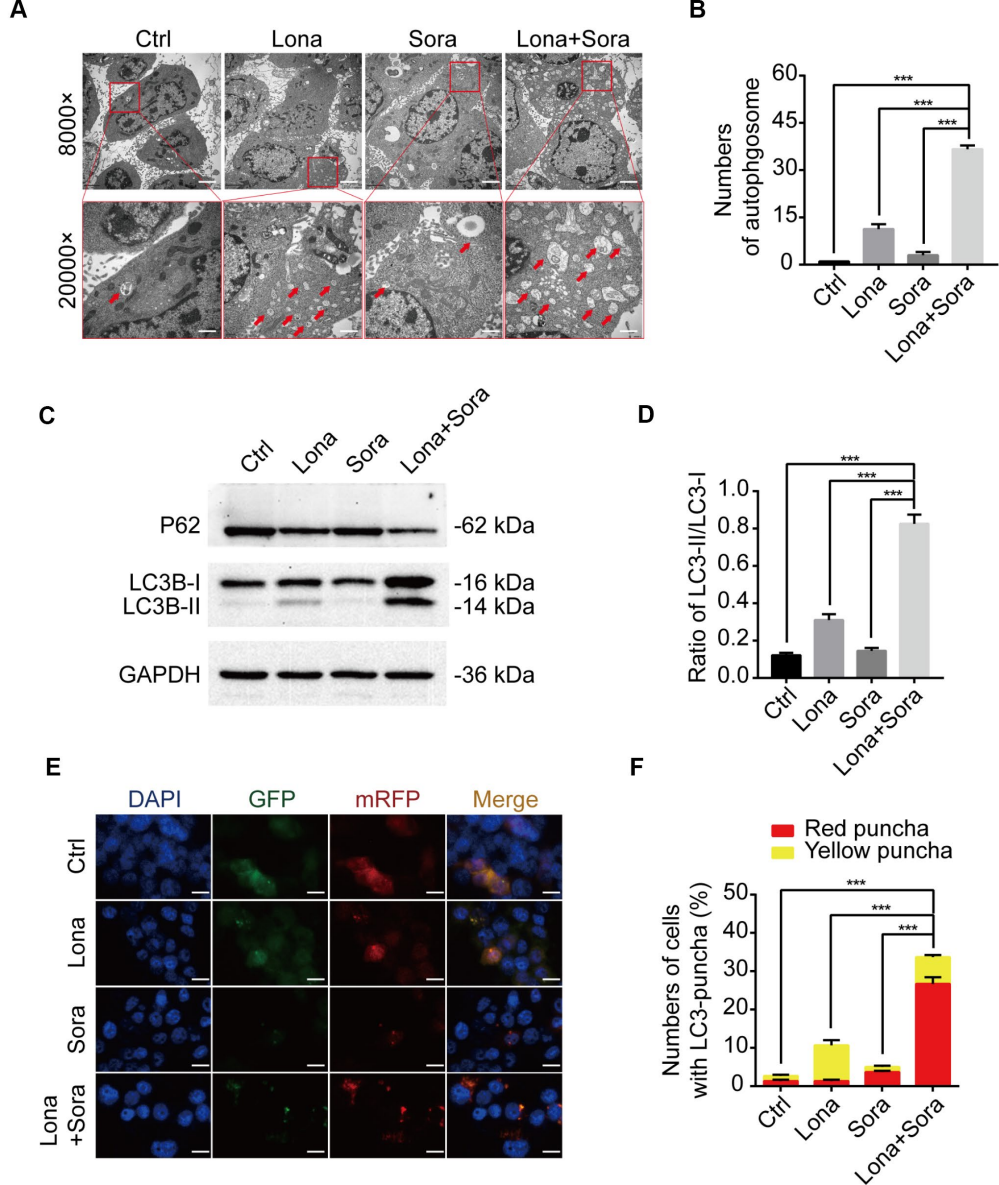
**Lonafarnib combined with sorafenib induces autophagic flux.** (**A**) Ultrastructural analysis showing autophagy induced by lonafarnib and sorafenib co-treatment in HepG2 cells. Red arrows indicated autophagosomes or autolysosomes. (**B**) The numbers of autophagosomes and autolysosomes were summarized and the data was presented as the means ± SD of triplicates. ***P < 0.001. (**C**) Western blot analysis of protein levels of P62 and LC3B. Cells were treated as indicated. (**D**) The ratio of LC3B-II/LC3B-I according to western blot results above. ***P < 0.001. (**E**) Detection of autophagic flux using mRFP-GFP-LC3 reporter in HepG2 cells after treatment with lonafarnib (10 μM) and/or sorafenib (5 μM) for 48 h. Microscopy images merged with GFP, RFP and DAPI fluorescence of representative cells. Scale bar = 10 μm. (**F**) The percentages of red (mRFP^+^ and GFP^-^, autolysosomes) and yellow (mRFP^+^ and GFP^+^, autophagosomes) were calculated. ***P < 0.001.

Previous reports have indicated that either lonafarnib or sorafenib is able to induce autophagy in cancer cells [23, 24]. Thus, we asked whether lonafarnib combined with sorafenib could induce autophagic flux in our cell model. After treatment with lonafarnib and/or sorafenib for 48 h, we observed an increased amount of typical autophagosomes with a double-membrane vesicular structure in HepG2 cells under transmission electron microscopy (Figure 3A and 3B). Consistent with this phenomenon, western blotting also confirmed the increased ratio of LC3-II/I protein expression and decreased protein level of P62, a cargo protein specifically degraded inside autolysosomes (Figure 3C and 3D). Similarly, we transfected HepG2 cells with a plasmid expressing mRFP/GFP-LC3 to monitor the autophagic flux status by fluorescence microscopy. The autophagic aggregates are indicated as intense yellow or red LC3 puncta without nuclear localization. In green/red merged images, yellow puncta (mRFP^+^ and GFP^+^) represents autophagosomes, while red puncta (RFP^+^ and GFP^-^) represents autolysosomes. Increased autophagic flux is defined when both yellow and red puncta are increased in cells, while blocked autophagic flux is defined when only yellow puncta are increased without an accompanying increase of red puncta in cells. Therefore, as shown in Figure 3E and 3F, in the presence of lonafarnib and/or sorafenib, accumulation of both yellow and red LC3 puncta was significantly increased in the combination treatment group compared with the single treatment group or control group (Figure 3E and 3F). These results confirm that lonafarnib combined with sorafenib strongly induces autophagic flux.

### ATG3 is involved in the autophagic flux induced by lonafarnib and sorafenib co-treatment

**Figure 4:**
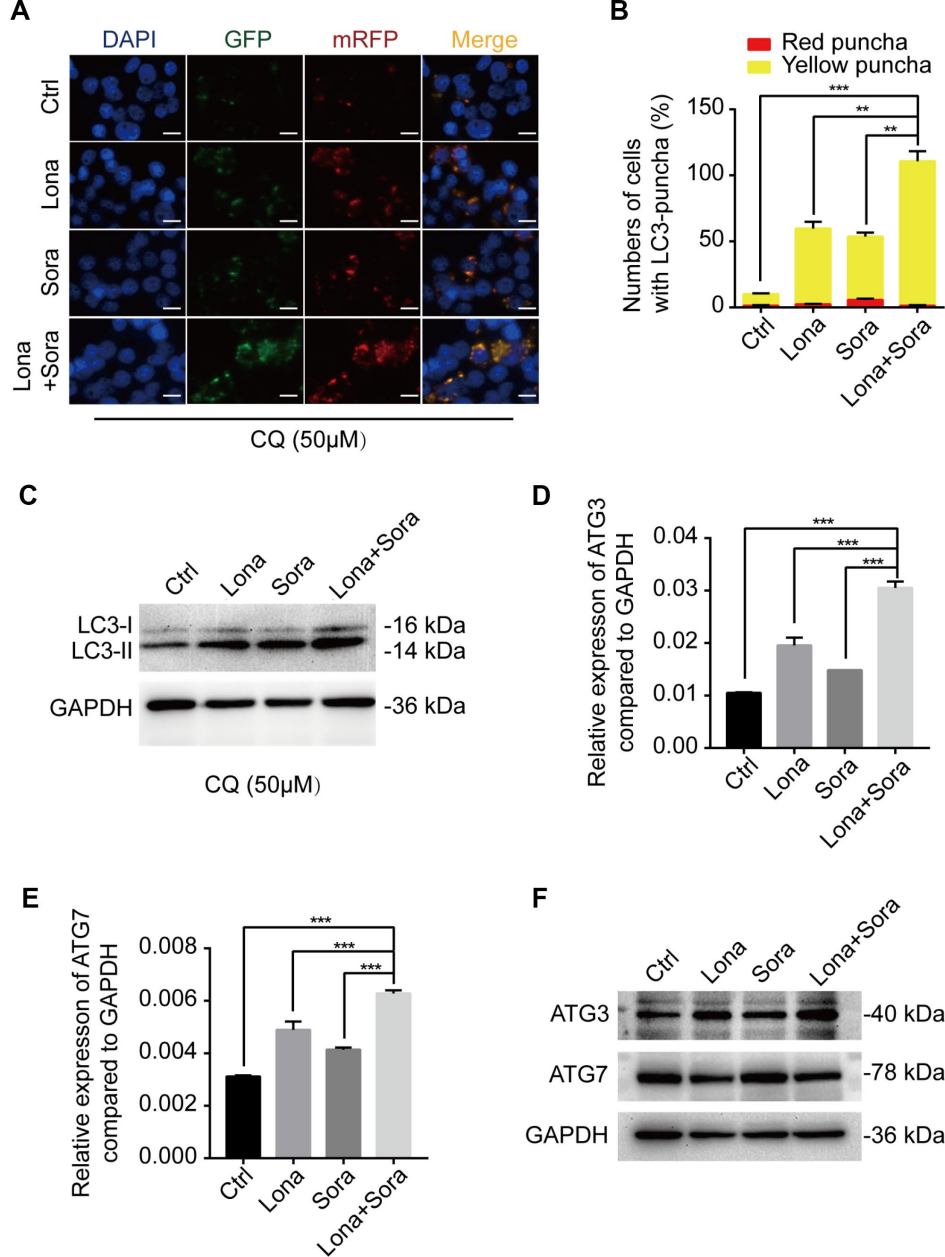
**ATG3 is involved in the autophagic flux induced by lonafarnib and sorafenib co-treatment.** (**A**) HepG2 cells were transfected with mRFP-GFP-LC3 reporter after treatment with lonafarnib (10 μM) and/or sorafenib (5 μM) plus CQ (50 μM) for 48 h. Microscopy images merged with GFP, RFP and DAPI fluorescence of representative cells. Scale bar = 10 μm. (**B**) The numbers of autophagosomes and autolysosomes were summarized and the data were presented as the means ± SD of triplicates. **P < 0.01; ***P < 0.001. (**C**) Western blot analysis of protein levels of LC3B. HepG2 cells were treated with lonafarnib and/or sorafenib in the presence of CQ (50 μM). (**D**) and (**E**) mRNA expression of ATG3 and ATG7 performed by qPCR assay. ***P < 0.001. (**F**) Western blot analysis of ATG3 and ATG7 protein. HepG2 cells were treated as indicated.

To further clarify the mechanism of lonafarnib and sorafenib co-treatment induced autophagic flux, we used the lysosome inhibitor, CQ, to block the degradation of autophagosomes and significantly promote the accumulation of the LC3 puncta. After combination treatment with lonafarnib and sorafenib, the amount of LC3 puncta increased approximately 2-fold compared to single drug treatment and 10-fold compared to control treatment (Figure 4A and 4B). Similarly, the expression level of LC3-II protein was also analyzed by western blotting. The LC3-II band was increased in the co-treatment group compared to other groups, indicating that co-treatment influenced the early stage of autophagosome formation (Figure 4C). Thus, q-PCR and western blotting assays were performed to assess the mRNA and protein expression levels of ATG3 and ATG7, two important proteins driving the conversion of LC3-I to LC3-II. An additive effect was observed in mRNA expression of ATG3 and ATG7 when HepG2 cells were exposed to combination treatment of lonafarnib and sorafenib (Figure 4D and 4E). However, only the protein expression of ATG3, but not ATG7, was unregulated in combination group (Figure 4F). These results suggest that ATG3 may be involved in the autophagic flux induced by lonafarnib and sorafenib co-treatment.

### Knockdown of ATG3 attenuates the autophagic flux induced by lonafarnib and sorafenib co-treatment

**Figure 5:**
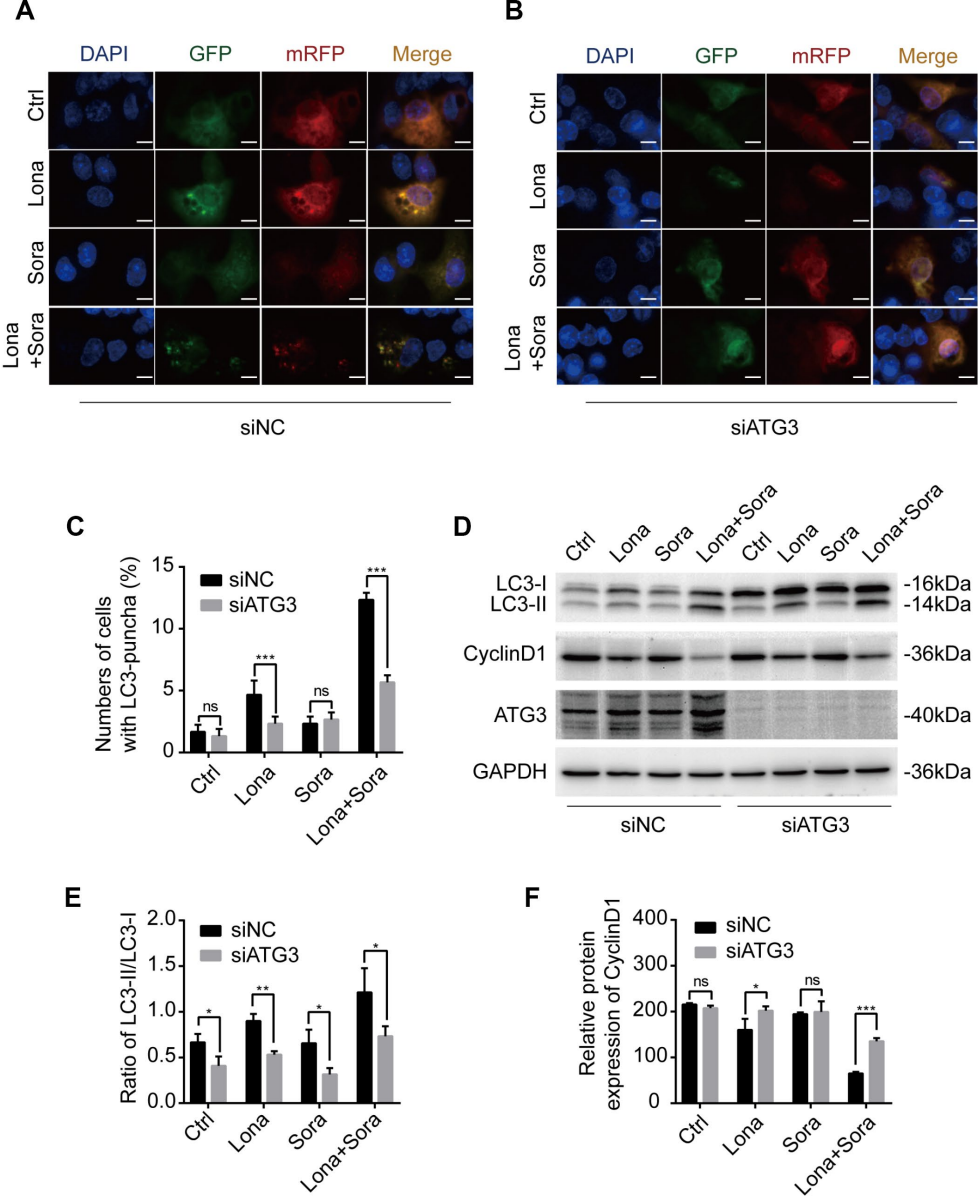
**Knockdown of ATG3 attenuates the autophagic flux induced by lonafarnib and sorafenib co-treatment.** (**A**) and (**B**) Detection of autophagic flux with the mRFP-GFP-LC3 reporter in HepG2 cells in response to siRNA treatments of negative control (NC) and ATG3 under a microscope. Scale bar = 10 μm. (**C**) Quantification of cells with LC3 puncta as indicated. ns, P > 0.05; ***P < 0.001. (**D**) Western blot analysis was performed to detect changes in LC3B and cyclin D1 proteins in cells transfected with indicated siRNAs. (**E**) and (**F**) Ratio of the conversion of LC3B-I to LC3B-II (**E**) and expression of cyclin D1 (**F**). ns, P > 0.05; *P < 0.05; **P < 0.01.

According to the above results, siRNA targeting ATG3 mRNA transcription was used to block the formation of autophagosomes. ATG3 depletion led to a heavy reduction of LC3 puncta in the combination treatment group and lonafarnib monotherapy group and had no effect on the sorafenib monotherapy group and control group (Figure 5A and 5B). Western blotting analysis also confirmed that compared to the negative control, accumulation of a larger amount of LC3-I was observed after knockdown of ATG3 expression in the combination group (Figure 5D and 5E). Autophagy is always closely related to cell cycle process, and cyclin D1 plays a key role in controlling G1/S phase transition [25]. Wu et al. reported that cyclin D1 was selectively degraded by autophagic flux [26]. Thus, we also tried to identify if lonafarnib and sorafenib co-treatment influenced the expression of cyclin D1. Western blotting analysis revealed that combination treatment markedly decreased cyclin D1 expression, while under ATG3 depletion condition, the reduction of cyclin D1 was partially abolished (Figure 5D and 5F). These results suggested that lonafarnib and sorafenib co-treatment induced autophagic flux and the accompanying cyclin D1 reduction in an ATG3-dependent pathway.

### Cyclin D1 is degraded in the autophagic flux induced by lonafarnib and sorafenib co-treatment

**Figure 6:**
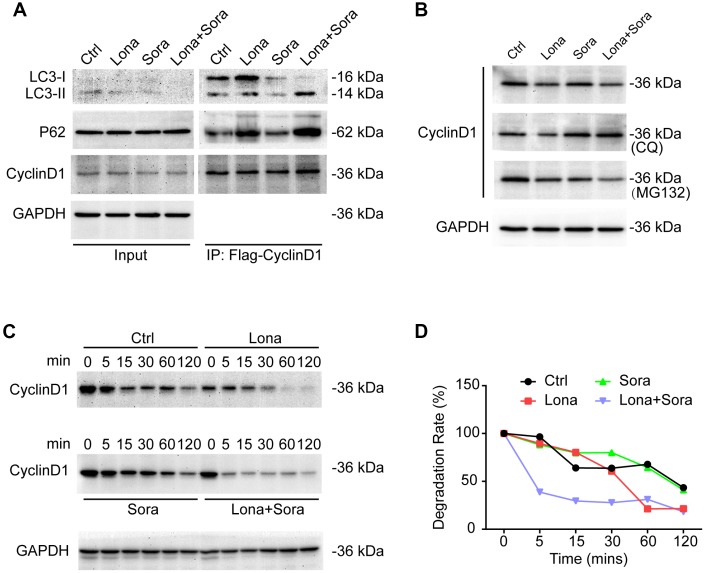
**Cyclin D1 is degraded in the autophagic flux induced by lonafarnib and sorafenib co-treatment.** (**A**) HepG2 cells were transfected with Flag-Cyclin D1 plasmid. Cell lysates were immunoprecipitated with anti-Flag agarose followed by immunoblotting to investigate the levels of LC3B, P62, and cyclin D1 proteins. (**B**) Cells were maintained in the presence of MG-132 or CQ for another 12 h after treatment with lonafarnib and/or sorafenib. Western blotting was used to detect changes in cyclin D1 protein expression. (**C**) HepG2 cells were treated as indicated, and western blot assay was used to detect cyclin D1 protein degradation. (**D**) The protein degradation rates of cyclin D1 were quantified in (**C**).

We next focused on clarifying whether cyclin D1 was degraded by autophagy in the presence of drug treatment. Cells were transfected with Flag-tagged cyclin D1 and autophagy was induced after treatment with lonafarnib and/or sorafenib for 24 h. Immunoprecipitation assay demonstrated that the interaction of cyclin D1 with both p62 and LC3-II (lapidated form) proteins was increased in the lonafarnib monotherapy group and much more so in the combination treatment group, suggesting that encapsulation of cyclin D1 protein in autophagosomes was increased after co-treatment (Figure 6A). In addition, the lysosome inhibitor CQ, but not the proteasome inhibitor MG-132, inhibited the decreased expression of cyclin D1 under co-treatment condition (Figure 6B). Furthermore, the cycloheximide chase experiment also confirmed that the degradation of cyclin D1 was markedly increased in the combination treatment group (Figure 6C and 6D). These results demonstrate that cyclin D1 is selectively degraded in the autophagic flux induced by lonafarnib and sorafenib co-treatment.

### Degradation of cyclin D1 caused by lonafarnib and sorafenib co-treatment inhibits DNA synthesis

**Figure 7:**
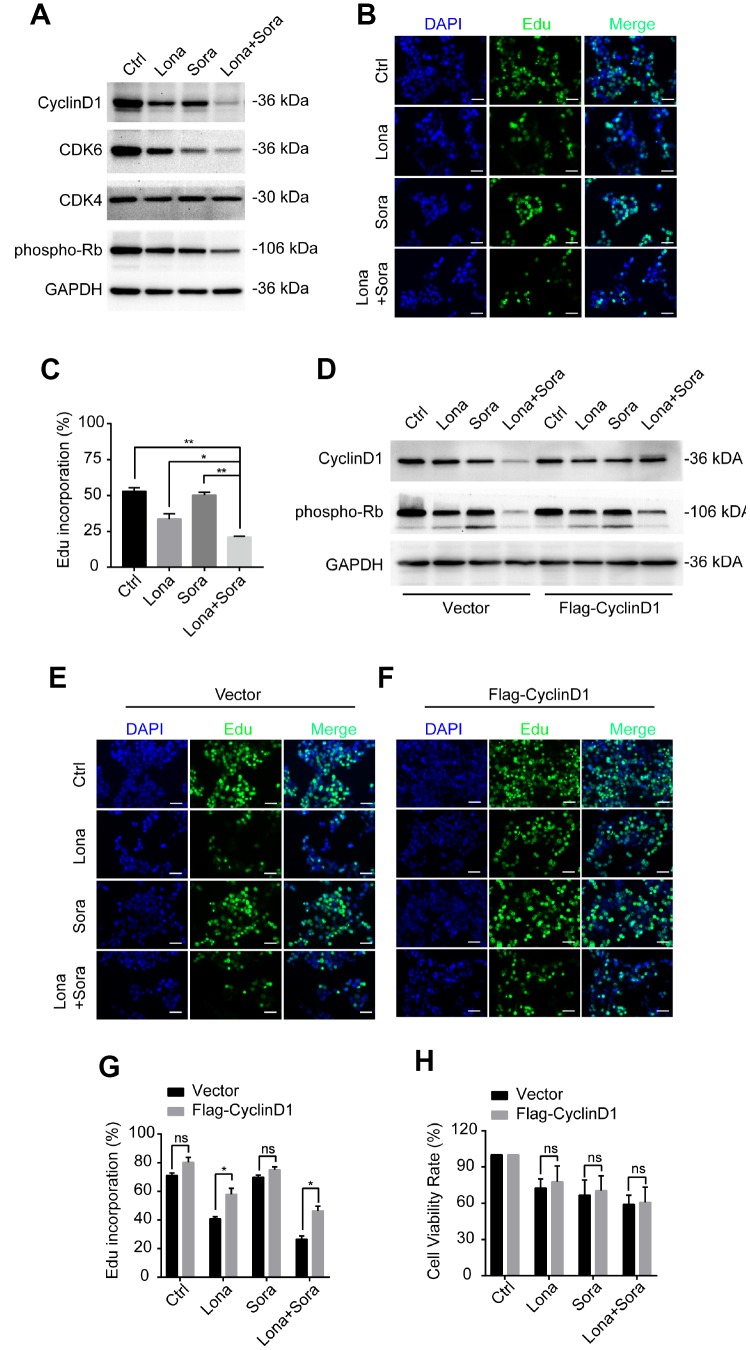
**Degradation of cyclin D1 caused by lonafarnib and sorafenib co-treatment inhibits DNA synthesis.** (**A**) HepG2 cells were treated with lonafarnib and/or sorafenib for 48 h. Protein expression levels were determined using the antibodies as indicated. (**B**) HepG2 cells were treated with lonafarnib and/or sorafenib for 48 h. DNA synthesis was analyzed by Edu staining. (**C**) Quantification of Edu positive straining in (**B**) was shown in the diagram.. *P < 0.05; **P < 0.01. (D) Protein expression levels of cyclin D1 and phospho-Rb in HepG2 cells transfected with vector control or Flag-Cyclin D1. (**E** and **F**) Representative image of Edu staining in HepG2 cells transfected with vector control or Flag-Cyclin D1. (**G**) Quantification of Edu positive straining in (**E**) and (**F**). ns, P > 0.05; *P < 0.05. (**H**) HepG2 cells were treated with lonafarnib and/or sorafenib for 48 h, and cell viability was evaluated by CCK-8 assay. ns, P > 0.05.

In addition to cyclin D1, we also analyzed the expression of other cell cycle-related proteins, including CDK4, CDK6 and phospho-Rb by western blotting. Compared to the monotherapy groups, cyclin D1, CDK6, and phospho-Rb, but not CDK4, were all decreased in the combined treatment group (Figure 7A). Since loss of the above-stated proteins may lead to cell cycle arrest at the G1/S phase transition, we further evaluated the DNA synthesis function after drug treatment. As shown in Figure 7B and 7C, Edu incorporation was significantly decreased after lonafarnib treatment and much more so after lonafarnib and sorafenib co-treatment. Forced expression of cyclin D1 in the presence of lonafarnib and sorafenib co-treatment markedly increased the expression of phospho-Rb protein level (Figure 7D) as well as the Edu incorporation level (Figure 7E–7G). However, cyclin D1 reconstitution did not alleviate the toxic effect of lonafarnib and sorafenib treatment even after the recovery of the DNA synthesis function (Figure 7H). Our results demonstrate that lonafarnib and sorafenib co-treatment inhibits DNA synthesis, which could be rescued by cyclin D1 expression.

## DISCUSSION

Unresectable and advanced HCC is highly refractory to conventional chemotherapy due to innate or acquired chemoresistance and adverse events. Currently, the multikinase inhibitor sorafenib is among the standard systemic therapy for the treatment of patients with advanced HCC [27]. The combination of sorafenib with compounds that target alternate signaling pathways may lead to new therapeutic options resulting in a decreased risk of metastasis and increased survival. FTase has been recently suggested as a drug target in the development of anti-cancer therapy [28]. FTase inhibitors were initially designed to block cell transformation by Ras protein whose activation promotes carcinogenesis via multiple signaling pathways. Although their antitumor abilities have been demonstrated with many cancers [29], the effect on HCC has received less attention. Different from our previous study in which we identified the synergistic effect of lonafarnib and sorafenib to inhibit the growth of HCC cell lines SMMC-7721 and QGY-7703 [30], in this study, using both *in vitro* and *in vivo* models, we confirmed the cytotoxic effect of this combination on another HCC cell line HepG2. Both lonafarnib and sorafenib alone were able to inhibit cellular proliferation, colony formation and induced cell death in HepG2 cells, and concurrent treatment with these two agents markedly increased the therapeutic effect. In addition, we demonstrated novel regulatory mechanism that combination treatment induced ATG3 dependent autophagic flux, leading to the degradation of cyclin D1 and subsequent growth inhibition. These results provide further support for the developing concept that simultaneous interruption of two relevant signaling pathways may act as a promising strategy for HCC therapy.

Autophagy is an intracellular degradative process targeting cytosolic components that maintains cell survival and supplies substrates for energy generation [31]. This process plays a dual role in HCC. On one hand, autophagy inhibits apoptosis of tumor cells and enhances tolerance to chemotherapy drugs by degrading damaged organelles and misfolding proteins to provide nutrients and energy for tumor survival. Toshima et al. reported that autophagy promotes cell proliferation, and liver undergoes rapid protein turnover for remodeling after partial hepatectomy [32]. Chen et al. revealed that peritumoral monocytes induce autophagy and activate NFκB-SNAI1 signaling pathway to promote tumor metastasis [33]. In sorafenib-resistant cells, overexpression of CD24 activates autophagy level by inhibiting mTOR/AKT pathway [34]. Zhang et al. reported that PU.1/microRNA-142-3p sensitizes HCC cells to sorafenib via inactivating ATG5/ATG16L1-mediated autophagy [35]. These findings indicated that increased autophagy may act as an oncogenic factor in HCC. On the other hand, excessive stimulation of autophagy may lead to autophagy-induced cell death due to overloaded self-consumption under continuous stress. Anti-tumor agent rapamycin and its derivatives induce autophagy by inhibiting the mTOR pathway. A recent study showed that overexpression of RASSF1 in HCC cells induced autophagy via inhibiting PI3K-AKT-mTOR signaling through the Hippo pathway module MST1 and suppressed HCC tumor growth [36]. Lin et al. showed that plumbagin induced both autophagy and apoptosis in SMMC-7721 cells *in vitro* and *in vivo* [37]. Thus, activation of autophagy may be beneficial in HCC treatment. In the present study, we demonstrated that the combination of lonafarnib and sorafenib promoted a strong activation of autophagic flux accompanied by growth suppression of tumor cells. Mechanistically, this co-treatment effect promotes the formation of autophagosomes by enhancing the transcription of ATG3 mRNA. Although these results were preliminary, they still provide evidence that autophagy induced by lonafarnib and sorafenib co-treatment contributes to regulation of HCC cell survival.

The G1/S phase transition is particularly critical in cell cycle, and once it is out of control, cells will evolve into malignant proliferation. Among the regulatory modules, cyclin D1-CDK4/6 complex and Rb protein have an important role in controlling cell cycle process. Cyclin D1 had been reported to be highly expressed in cancers of various origins, such as breast, esophageal, colon, and bladder. Knockdown of cyclin D1 expression shortens the G1 phase and causes G1/S phase arrest. Currently, cyclin D1 has been identified as an oncogene in HCC. Overexpression of cyclin D1 enhanced liver cell growth, colony formation, and accelerated HCC development by promoting cell cycle progression [17]. Under normal conditions, cyclin D1 is degraded by the ubiquitin-proteasome system. A variety of drugs targeting cyclin D1 degradation have been extensively studied. Langenfeld J et al. demonstrated that all-trans retinoic acid decreased cyclin D1 expression and extended G1/S transition [38]. In prostate cancer, breast cancer and squamous carcinoma cells, curcumin not only downregulated cyclin D1 mRNA expression, but also promoted cyclin D1 proteolysis to exert its antiproliferation activity [39]. In addition, in SW480 cells, both resveratrol and aspirin specifically decreased cyclinD1 expression and induced cell death. The decrease in cyclin D1 can be blocked by proteasome inhibitors, calpain inhibitor I and MG-132 [40, 41]. Thus, reducing cyclin D1 expression may be an effective way for HCC therapy. In our study, we observed a marked decrease in cyclin D1 induced by lonafarnib and sorafenib treatment that was blocked by CQ but not MG-132, indicating that this degradation was achieved through a lysosome-dependent way. Further study confirmed that cyclin D1 was degraded by drug-induced autophagy process, which was consistent with Wu’s research [26]. Taken together, our results revealed that the combination of lonafarnib and sorafenib induced cyclin D1 degradation in an autophagy-dependent manner that suppressed HCC cell proliferation.

Although the combination of low dosages of lonafarnib and sorafenib is still toxic to normal cells, it is more sensitive to HCC cells. The toxic side effects are expected to be optimized by means of drug-delivery methods [42]. In addition, co-treatment induced cyclin D1 degradation and accompanying DNA synthesis block. Reconstitution of cyclin D1 rescued the DNA synthesis function but not the revival of cell viability. Since cell survival relies on the concerted adjustment of various signals, autophagic flux induced degradation of cyclin D1 may be one of the “passenger phenomena”, but not the major mechanism accounting for viability maintenance by combination treatment of lonafarnib and sorafenib in HCC cells. Further studies are needed to clarify the underlying mechanism.

**Figure 8:**
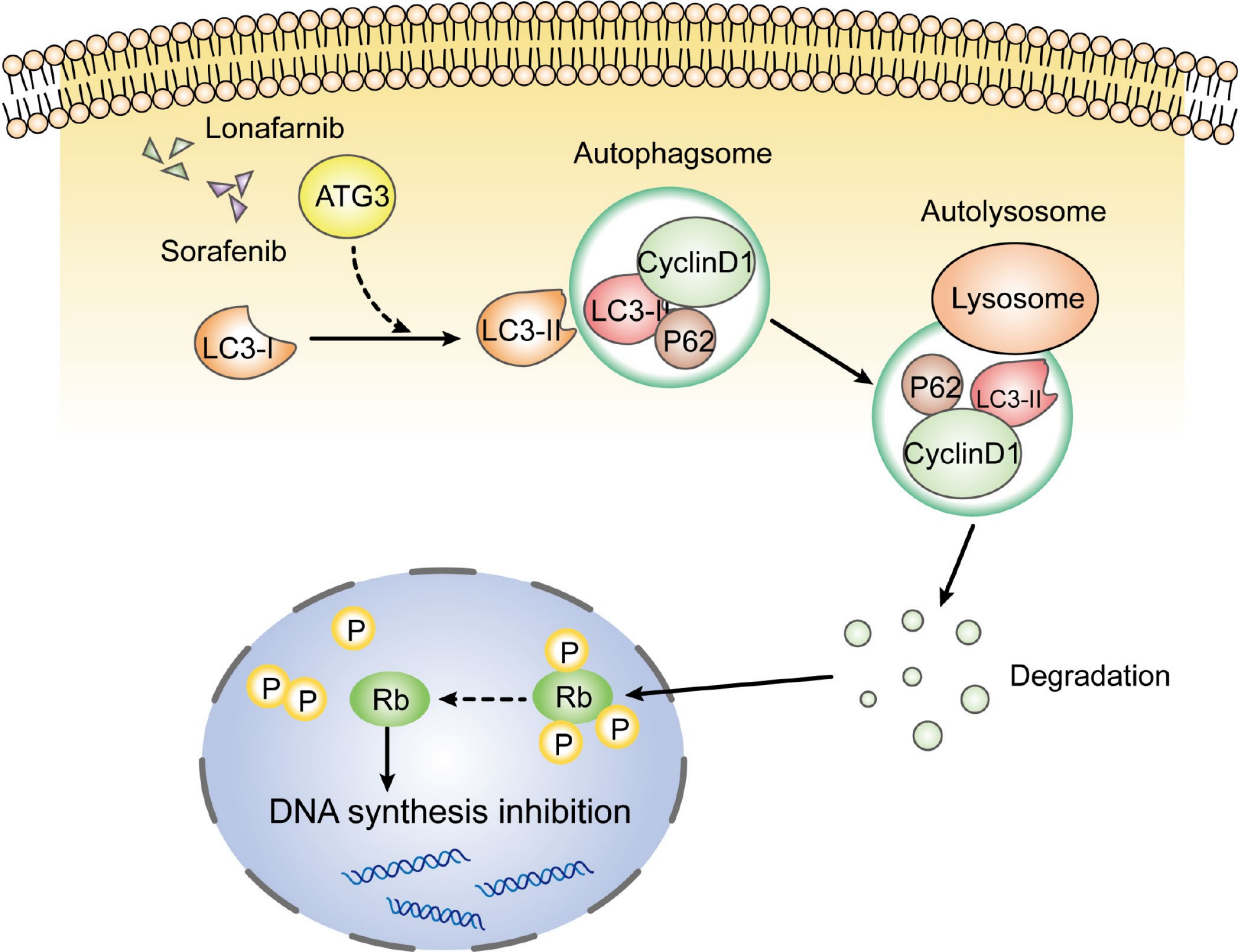
**Schematic model of this study.** After the combination treatment of lonafarnib and sorafenib, ATG3 protein was increased and facilitated the conversion of LC3-I to LC3-II, which activates the autophagic flux that recruits cyclin D1 protein into autolysosome and promotes its degradation. The lack of cyclin D1 leads to hypophosphorylation of Rb protein, and subsequent inhibition of DNA synthesis.

In conclusion, our results demonstrate that the combination of lonafarnib and sorafenib induced the formation of autophagosomes and suppressed cell viability, and a decrease in cyclin D1 by autophagic flux and subsequent cell cycle arrest were involved in the mechanism mediating the effect of lonafarnib and sorafenib co-treatment in HCC cells Figure 8.

## MATERIALS AND METHODS

### Reagents

Lonafarnib (S2797), sorafenib (S7397), chloroquine (CQ, S4157), MG-132 (S2619) were obtained from Selleck Chemicals (TX, USA). Stock solution of 10 mM lonafarnib, 20 mM sorafenib, 100 mM chloroquine and 100 mM MG-132 were dissolved in dimethyl sulfoxide (DMSO, Sigma-Aldrich) and stored at −80°C. Cell Counting Kit-8 (CCK8) was purchased from Dojindo Molecular Technologies Inc. (Kumamoto, Japan). The mRFP-GFP-LC3B adenovirus construct was purchased from Hanbio Inc. (Shanghai, China). Cell-Light Edu Apollo488 In Vitro Kit was purchased from RiboBio Company (Guangzhou, China). Antibodies used were as follow: P62 (#88588, Cell Signaling Technology), ATG3 (#3415, Cell Signaling Technology), ATG7 (#8558, Cell Signaling Technology), cyclin D1 (#2978, Cell Signaling Technology), CDK4 (#12790, Cell Signaling Technology), CDK6 (#13331, Cell Signaling Technology), phosphor-Ser780-Rb (#8180, Cell Signaling Technology), LC3B (GTX127375, GeneTex), GAPDH (60004-1-1g, Proteintech).

### Cell cultures

The human HCC cell line HepG2 and immortalized hepatic cell line MIHA were obtained from the Third Affiliated Hospital of Sun Yat-sen University. All cell lines were cultured in DMEM containing 10% FBS in a 5% CO_2_ incubator at 37°C and passaged every two days. All cell lines were thawed from early passage stocks.

### Plasmid construction and transfection

The cDNA encoding full-length human CCND1 was amplified by PCR from cDNA library of 293T cells and subcloned into pcDNA3.1(+) vector (Invitrogen). During the PCR, the Flag tag (DYKDDDDK) was added to the N-terminus of the indicated protein. Constructed Flag-Cyclin D1 plasmid was transfected into HepG2 cells using Lipofectamine 2000 (Invitrogen, Carlsbad, CA, USA) for 48 h. Targeting ATG3 siRNA duplexes (siATG3 sequence: GCTCAGCACTATGTGAAGA) were obtained from RiboBio Company (Guangzhou, China). HepG2 cells were transfected with 100 nM siRNA using Lipofectamine RNAiMAX according to the manufacturer’s instructions (Invitrogen, Carlsbad, CA, USA).

### CCK-8 assay

Cell viability was assessed with CCK-8 assay. A total of 3×10^3^ cells were seeded in 96-well plates and incubated at 37°C for 24 h to adhere. Different concentrations of lonafarnib and/or sorafenib were added into the culture medium and incubated for either 24 or 48 h. At the end of these periods, 10 μl of CCK-8 reagent was added to each well. After 4 h, the absorbance (OD value) at 450 nm was measured with a spectrometer (SpectraMax M5 Microplate Reader, Molecular Devices LLC). The IC50 of two agents was determined using GraphPad Prism 7.0 software.

### Western blotting

HepG2 cells were seeded in 6-well plates and given treatments as described. At approximately 80% confluence, cells were lysed in NETN buffer (20 mM Tris-HCl at pH 8.0, 100 mM NaCl, 1 mM EDTA, 0.5% Nonidet P-40) containing protease and phosphatase inhibitor cocktails (Thermo Fisher, USA). The protein concentration was determined by the BCA protein assay kit (Pierce). After normalization, protein extracts were subjected to 10% or 15% SDS-PAGE, transferred to the PVDF membrane (Bio-Rad Laboratories, USA) and probed with the indicated primary antibodies at 4°C overnight. The blots were incubated with species-specific HRP-conjugated secondary antibodies. Enhanced chemiluminescence (ECL, Pierce) was used for detection. Relative band intensities were determined by quantification of each band using ImageJ software.

### Colony formation assay

A total of 1,000 HepG2 cells were seeded in 6-well plates and allowed to attach overnight and maintained in fresh medium with lonafarnib and/or sorafenib at 37°C. After 2 weeks, the medium was removed, and colonies were fixed with precooling methanol for 15 min prior to crystal violet (C01201, Beyotime) staining. Colony counting was performed using ImageJ software.

### Terminal deoxynucleotidyl transferase-mediated dUTP Nick End Labeling (TUNEL) assay

The DNA fragmentation of HepG2 cells was detected using TUNEL assay. In total, 1×10^5^ cells were seeded in 48-well plates and maintained in medium with lonafarnib and/or sorafenib. After 48 h, cells were fixed with formaldehyde for 15 min and then incubated in 0.1% Triton-100 for 5 min at room temperature. DNA labeling solution was added into the medium for 60 min at 37°C, and the DNA fragmentation of HepG2 cells was detected with fluorescence microscopy and analyzed using ImageJ software.

### Animal studies

All mice procedures were done according to a protocol approved by the Institutional Animal Care and Use Committee of Third Affiliated Hospital of Sun Yat-sen University, and handled in accordance with the Guide for the Care and Use of Laboratory Animals. Female nude mice aged 4-6 weeks were purchased from Charles River Laboratories (Beijing, China) and housed in a defined pathogen-free environment. One week later, all nude mice were injected subcutaneously with HepG2 cells (1 × 10^7^) into the right armpit. When tumor volumes reached approximately 100 mm^3^, nude mice were randomly assigned to 4 groups. Group 1 (control) received 20% hydroxyl-propyl-betacyclodexatrin (20% HPβCD, Sigma-Aldrich) and Cremophor EL (Sigma-Aldrich)/95% ethylalcohol mixture (Cremophor EL:95% ethylalcohol = 50:50). Group 2 (lonafarnib group) received 20 mg/kg lonafarnib (dissolved in 20% HPβCD) twice daily. Group 3 (sorafenib group) received 40 mg/kg sorafenib (dissolved in Cremophor EL/95% ethylalcohol mixture) once daily. Group 4 (combination group) received20 mg/kg lonafarnib (twice daily) plus 40 mg/kg sorafenib (once daily). Drugs were administered by oral gavage. Tumor size was measured every 3 days. At the endpoint of this experiment, mice were humanely killed using cervical dislocation, and tumors from 4 groups were isolated and weighed. The rates of tumor growth were quantified by measuring tumor volume in perpendicular diameters and the volume of tumors was calculated using the formula: tumor volume= π/6 × large diameter × smaller diameter^2^.

### RNA isolation and real-time quantitative PCR

Total RNA from cultured cells was isolated with TRIzol reagent (Invitrogen) following the manufacturer’s instructions. cDNA was synthesized from 2 μg of purified RNA with random primers using GoScript^TM^ Reverse Transcription System (Promega). Real-time quantitative PCR was performed with Platinum SYBR Green qPCR SuperMix-UDG (Invitrogen) on a LightCycler 480 PCR platform (Roche). The expression of relative mRNAs was assessed based on the threshold cycle (Ct). Normalized expression was calculated as 2^ΔΔCt^ to GAPDH expression. The sequences of the primers used are as follows: ATG3 (F: GACCCCGGTCCTCAAGGAA, R: TGTAGCCCAT TGCCATGTTGG), ATG7 (F: CAGTTTGCCCCTTTTA GTAGTGC, R: CCAGCCGATACTCGTTCAGC), GAP DH (F: TGTGGGCATCAATGGATTTGG, R: ACACC ATGTATTCCGGGTCAAT).

### mRFP-GFP-LC3 reporter assay

Autophagic flux was determined by mRFP-GFP-LC3 reporter assay according to the manufacturer’s instructions. This assay was designed based on the different sensitivities of GFP and RFP to acidic environments. Both GFP and RFP are detected in autophagosomes, present as yellow puncta. Because GFP fluorescence is much more sensitive to the acidic environment in autolysosomes than mRFP fluorescence, once autophagosomes fuse with lysosomes, GFP fluorescence is lost due to the degradation of GFP by acid lysosome proteases, resulting in only the detection of RFP fluorescence, which is indicative of autolysosomes. Therefore, the switch in colors suggested a different period of autophagic flux. Autophagic flux was determined by quantifying the percentage of cells considered as LC3 positive puncta.

### Immunoprecipitation

HepG2 cells were harvested in lysis buffer after transfection with plasmid encoding Flag-cyclin D1. Flag-agarose beads were mixed with the sample lysates at 4°C for 6 h. Agarose beads were then washed five times with NETN buffer and immunoprecipitates were collected by boiling beads in 50 μl of 1×SDS sample buffer for 15 min. Finally, the supernatant was subjected to SDS-PAGE and western blotting analysis.

### Edu staining assay

HepG2 cells were seeded in 12-well plate and treated with lonafarnib and/or sorafenib for 48 h. The cells were incubated in medium containing 50 μM Edu for 30 min and fixed in acidic ethanol at room temperature. After 30 min, cells were incubated in 0.5% Triton X-100 for 10 min. Anti- Edu polyclonal antibody at a dilution of 1:400 was added to the well at room temperature for 1 h, and the cells were stained under a fluorescence microscope.

### Transmission electron microscopy

HepG2 cells were harvested and fixed in 2% glutaraldehyde with 2% paraformaldehyde in 0.1 M cacodylate buffer (pH 7.3) for 1 h. Samples were then washed using 1% cacodylate-buffered tannic acid, postfixed in 1% uranyl acetate before dehydration in ethanol, embedment in Spurr’s low-viscosity embedding medium, and polymerization at 60°C for 2 days. Ultra-thin sections of the cells were stained with uranyl acetate and lead citrate and analyzed under a JEM-1010 transmission electron microscope (JEOL, Tokyo, Japan).

### Statistical analysis

SPSS software version 19.0 and GraphPad Prism 7.0 were used to perform the statistical analysis. The results are expressed as the mean ± standard deviation, and represent the average values from 2-3 values/experiments. The significance of variance between groups was determined by Student’s t-test. All statistical tests were two-sided, and P < 0.05 was considered statistically significant.
