# Correction for: The combination of lonafarnib and sorafenib induces cyclin D1 degradation via ATG3-mediated autophagic flux in hepatocellular carcinoma cells

**DOI:** 10.18632/aging.102289

**Published:** 2019-09-12

**Authors:** Jialiang Wang, Huan Wei, Yanlin Huang, Dongmei Chen, Guofen Zeng, Yifan Lian, Yuehua Huang

**Affiliations:** 1 Guangdong Provincial Key Laboratory of Liver Disease Research, The Third Affiliated Hospital of Sun Yat-sen University, Guangzhou, China; 2 Department of Infectious Diseases, The Third Affiliated Hospital of Sun Yat-sen University, Guangzhou, China

**Keywords:** correction

**This article has been corrected:** The authors made a mistake in the grant number in Funding. The authors declare that this correction does not change the results or conclusions of this paper. The authors sincerely apologize for this error. The correct grant number of Guangdong Provincial Key Research and Development Program is marked in bold font below.

Original article: Oncotarget. 2019; 11:5769–5785. 5769-5785 . https://doi.org/10.18632/aging.102165

## FUNDING

This study was supported in part by National Natural Science Foundation of China (grant number 81872006), Guangdong Provincial Key Research and Development Program (grant number **2019B110233002),** Major Project of Collaborative Innovation of Guangzhou Science and Technology Program (grant number 201704020175) and Natural Science Foundation of Guangdong Province (grant number 2018A030313592).

